# Hemodynamic Response to Laryngoscopy and Intubation Using McCoy Laryngoscope: A Descriptive Cross-sectional Study

**DOI:** 10.31729/jnma.6752

**Published:** 2021-06-30

**Authors:** Gajal Lakhe, Suresh Pradhan, Santosh Dhakal

**Affiliations:** 1 Department of Anesthesia, Manipal College of Medical Sciences, Phulbari, Pokhara, Nepal

**Keywords:** *hypertension*, *intubation*, *laryngoscopy*

## Abstract

**Introduction:**

Laryngoscopy and intubation are stressful as they lead to a rise in heart rate and blood pressure. Though transient, it may be detrimental to the cardiac and neurosurgical patients. There is a need to explore the possibility of obtunding the pressor response to laryngoscopy and intubation with the use of McCoy blade laryngoscope. We aimed to find out the hemodynamic response to laryngoscopy and intubation using McCoy laryngoscope in adult patients undergoing general anesthesia.

**Methods:**

The descriptive cross-sectional study was conducted in 37 American Society of Anesthesiologists' Physical Status I/IIpatients, with normal airway from December 2019-May 2020 in a tertiary care hospital. Ethical approval was obtained from Institutional Research Committee (reference number.: MEMG/IRC/290/GA). Convenience sampling method was used. The mean systolic and diastolic blood pressures were measured at baseline, one, three and five minutes after laryngoscopy and intubation. Data were analyzed using the Statistical Package for the Social Sciences Version 21.0.

**Results:**

In the first minute after laryngoscopy and intubation, the rise in mean blood pressure was noted in 14 (37.83%) cases. The peak rise in mean blood pressure was 3%, note done minute after laryngoscopy and intubation.

**Conclusions:**

We noted better attenuation of pressor response to laryngoscopy and intubation using McCoy blade laryngoscope in adult patients undergoing general anesthesia.

## INTRODUCTION

The hemodynamic response to laryngoscopy and intubation is inevitable, transient and mostly well tolerated by healthy adults. It is a reflex phenomenon mediated by Vagus(X) and glossopharyngeal (IX) nerve which carry afferent signal from epiglottis and infraglottic region and activate vasomotor center to cause peripheral sympathoadrenal response leading to hypertension, tachycardia and elevated serum catecholamines.^1-4^

The objective of the study was to focus on the non-pharmacological technique for the obtundation of pressor response to laryngoscopy and intubation by using McCoy blade laryngoscope. The wide array of drugs and various non pharmacological techniques have been tried to attenuate the hemodynamic response to laryngoscopy and intubation.^5-12^

We aimed to study the change in mean, systolic and diastolic blood pressure after laryngoscopy and intubation using McCoy laryngoscope in adult patients undergoing elective surgeries under general anesthesia.

## METHODS

The descriptive cross-sectional study was conducted in the department of Anesthesia of Manipal Teaching Hospital, Pokhara, Nepal from December 2019-May 2020. Ethical approval was taken from Institutional Review Committee (referencenumber: MEMG/IRC/290/ GA). The written and informed consent was taken from all patients enrolled in study. Convenient sampling technique was used. Sample size was calculated by using the formula given below:

$$\begin{array}{ll} \text{n} & \text{= }{\text{Z}}^{\text{2}}\times \text{p}\times \text{(1}-\text{p)}/{\text{e}}^{\text{2}}\\ & \text{= }{\left(1.96\right)}^{\text{2}}\times 0.5\times (1-0.5)/{(\text{0.05)}}^{\text{2}}\\ & \text{= }385 \end{array}$$

Since, there were a total of 40 patients underwent general surgery who required endotracheal intubation in 6 months duration, adjusting the sample size for finite population,

n° = n x N / {(N + (n-1)}

  = 37

where,

n = minimum required sample size
Z = 1.96 at 95% Confidence Interval (CI)
p = prevalence taken as 50% for maximum sample size
q = 1-p
e = margin of error i.e., 0.05
n° = adjusted sample size
N = total number of patients undergoing general surgery requiring endotracheal intubation, 40

A total of 37 adults (18-60 years) of both gender, American Society of Anesthesiologists (ASA) physical status I/II, who underwent elective general surgery under general anesthesia requiring endotracheal intubation were enrolled.

Patients with anticipated difficult intubation (Mallampati Class III and IV, thyromental distance <6 cm, inter-incisor distance <3 cm, and cervical instability), hemodynamically unstable patients, patients withhypertension, ischemic heart disease, cerebrovascular disease, heart block, heart failure, previous difficult intubation, severe respiratory distress, patients on β-blockers, vasodilators,patients who required second attempt for intubation, duration of laryngoscopy and intubation exceeding 30 seconds were excluded from study.

Pre anesthetic evaluation was done for all patients. All the patients were pre-medicated with tablet ranitidine 150 mg night before surgery and on the morning of surgery. Sedative premedication was not given.

The venous access was secured with 18G iv cannula on the dorsum of hand in pre-operative holding area. After shifting patients to operation room, pre induction ASA standard monitors electrocardiogram (ECG), noninvasive blood pressure (NIBP) and oxygen saturation (spo2) were attached. Baseline heart rate (HR), systolic blood pressure (SBP), diastolic blood pressure (DBP) and mean arterial pressure (MAP) werenoted. Ringer's lactate was started at 10 ml/kg. A doughnut-shaped pillow was placed under the head of patient to obtain classical sniffing position. General anesthesia was standardized for all cases. After three minutes of pre-oxygenation with 100% oxygen, anesthesia was induced with fentanyl 2 μg/kg, propofol 2-2.5 mg/kg and maintained with 60% nitrous oxide in oxygen and isoflurane 1% inspired. Neuromuscular blockade was achieved using succinylcholine 1-1.5 mg/kg. After 60 seconds of assisted ventilation laryngoscopy and intubation was performed with head of the patient in sniffing position. The stylet, boogie and external laryngeal manipulation was not used in any cases. The trachea was intubated with size 7 and 8 polyvinylchloride endotracheal tube in female and male patients, respectively. All the laryngoscopy and intubation were performed by the first author.

The hemodynamic variables: HR, SBP, DBP and MAP were recorded at baseline, one, three and five minutes after intubation. Duration of laryngoscopy and intubation was recorded. Duration of laryngoscopy and intubation was defined as the time starting from insertion of laryngoscope into patient's oral cavity to insertion of endotracheal tube into trachea. This time was recorded by anesthesia resident using stopwatch. The possible complications such as arrhythmias or ST segment changes (elevation or depression) were noted and managed accordingly.

Data analysis was done in Statistical Package for the Social Sciences (SPSS Inc., Chicago, IL, version 21.0 for windows). Descriptive statistics were performed and results expressed as mean±SD and frequency and percentage wherever applicable.

## RESULTS

A total of 37 patients were enrolled in study. In the first minute after laryngoscopy and intubation, the rise in mean blood pressure was noted in 14 (37.83%) cases. Likewise, the rise in systolic and diastolic blood pressure were noted in 13 (35.13%)and 15 (40.54%) cases respectively. All three parameters have increased in the first minute after laryngoscopy and intubation (Table 1).

**Table 1:** Hemodynamic parameters at various time interval (n=37).

Parameters	Tb§	Ti||	T3¶	T5**
MAP* (mmHg)	95.46±9.68	98.08±19.91	89.81±14.21	82.73±10.67
SBP† (mmHg)	125.05±11.63	126.89±22.37	119.68±18.45	109.89±12.71
DBP‡ (mmHg)	76.97±9.64	79.70±18.62	70.78±13.77	64.59±9.87

* MAP: mean arterial pressure

† SBP: systolic blood pressure

‡ DBP: diastolic blood pressure

§ Tb: baseline

|| T1: at one minute

¶ T3: at three minutes

** T5: at five minutes, data presented as mean±SD.

The rise was noted in the first minute post laryngoscopy and intubation after which it gradually decreased to below the baseline values (Table 2).

**Table 2:** Changes in blood pressures from baseline (n=37).

Parameters	Change in %
MAP^*^	
T1§	3%
T3||	-6%
T5¶	-13%
SBP†	
T1	2%
T3	-4%
T5	-12%
DBP‡	
T1	4%
T3	-7%
T5	-15%

* MAP: mean arterial pressure

† SBP: systolic blood pressure

‡ DBP: diastolic blood pressure

§ T1: at one minute

|| T3: at three minutes

¶ T5: at five minutes, data presented as mean±SD

The demographic features of patients enrolled in the study are presented (Table 3). The mean age and BMI of the patients enrolled in the study were 34.05±13.08 and 24.75±6.01 respectively. The mean duration of laryngoscopy and intubation was 15.11±7.26 seconds. A total of 32 (86.5%) cases belonged to American Society of Anesthesiologists Physical Statusgrade I and 5 (13.5%) cases belonged to grade II. Out of 37 cases 10 (27%) were males and 27 (73%) were female (Table 3).

**Table 3:** Demographic Variables (n=37).

Parameters	Mean±S.D.
Age (years) (mean±SD)	34.05±13.08
BMI^*^ (kg/m^2^) (mean±SD)	24.75±6.01
DLI^†^ (mean±SD)	15.11±7.26
Variable	n (%)
ASA PS^‡^	
I	32 (86.5)
II	5 (13.5)
Gender	
Male	10 (27)
Female	27 (73)

* BMI: Body Mass Index

† DLI: duration of laryngoscopy and intubation

‡ ASA PS: American Society of Anesthesiologists Physical Status.

## DISCUSSION

In our study we noted better attenuation of pressor response to laryngoscopy and intubation with McCoy laryngoscope. Our findings are similar to other studies.^11,13-15^ The force exerted by laryngoscope at base of tongue while lifting epiglottis is responsible for circulatory response to laryngoscopy and intubation. Direct laryngoscopy and intubation can lead to increase in heart rate and blood pressure by 20-27% and 30-50% respectively.^16^ In our study, the changes in hemodynamic parameters were within 20% from baseline which was clinically insignificant.

Our study is also in accordance with study conducted by Singhal et al., where they confirmedless circulatory response withuse of McCoy blade laryngoscope.^8^

The hemodynamic response is directly proportional to force exerted to expose glottis.^9^ As there is less mechanical stimulation with use of McCoy blade laryngoscope, lesser hemodynamic response is elicited which is also proved by our study.

The hemodynamic response to laryngoscopy and intubation is proportional to the duration of laryngoscopy and intubation beginning at 15 seconds and peaking at 45 seconds and lasts five minutes. As all the laryngoscopy and intubation were done by an experienced anesthesiologist, time taken to intubate was less than 30 seconds in all cases. No intubation aids including external laryngeal manipulation, use of stylet was needed in any of the patients due to easy visualization of glottis. This finding is similar to past study.^17^ We believe that shorter time to laryngoscopy and intubation and avoiding the use of intubation aids also led to favorable hemodynamic outcome in our study.

Our study has some limitations. First, our study was conducted in ASA I-II patients with normal airway; therefore, hemodynamic response may be exaggerated in patients with hypertension or in cases of difficult airway. Further research in such scenario is highly suggested. The invasive blood pressure monitoring would have been ideal, but it was avoided due to ethical considerations. Also, this study had been done in a single center with a limited sample size.

## CONCLUSIONS

We noted better attenuation of pressor response to laryngoscopy and intubation using McCoy laryngoscope in adult patients undergoing elective surgeries under general anesthesia.
